# Geographic disparities in physical activity, sedentary behavior, sleep time, and gross motor skills in Nigerian preschoolers

**DOI:** 10.3389/fpubh.2025.1506705

**Published:** 2025-03-13

**Authors:** John Oginni, Oluwayomi Aoko, Ekundayo Ajiborisade, Chukwuebuka Osuji, Anthony D. Okely, Kar Hau Chong, Penny L. Cross, Zan Gao

**Affiliations:** ^1^Department of Kinesiology, Recreation, and Sport Studies, The University of Tennessee, Knoxville, TN, United States; ^2^Department of Human Kinetics and Health Education (Exercise Physiology Unit), University of Lagos, Lagos, Nigeria; ^3^Department of Kinesiology and Dance, New Mexico State University, Las Cruces, NM, United States; ^4^School of Social Sciences, Early Start, Faculty of the Arts, Social Science and Humanities, University of Wollongong, Wollongong, NSW, Australia; ^5^Research Services Office, Research and Sustainable Futures Division, University of Wollongong, Wollongong, NSW, Australia

**Keywords:** accelerometry, movement behaviors, sleep, lower middle-income country, kindergarten

## Abstract

**Background:**

Adherence to physical activity guidelines is crucial for children’s physical, social, and cognitive well-being. While previous research shows that about 77% of preschoolers meet the World Health Organization (WHO) physical activity guidelines, differences in outcomes based on geographic locations are unclear. This study examines disparities in physical activity, sedentary behavior, sleep, and gross motor skills between rural and urban preschoolers.

**Methods:**

The study involved 131 healthy preschoolers (3.4 ± 0.8 years) from Lagos State, Nigeria. Participants wore ActiGraph GT3X+ accelerometers on their hips for at least 72 h per week. Socio-demographic data were collected using a parent-reported survey. Physical activity (light, moderate-vigorous) and sedentary time were measured using accelerometers, while gross motor skills were assessed using a modified version of the NIH Toolbox. Independent sample t-tests tested the difference between the two groups.

**Results:**

No significant difference in sleep time (t = −0.22, *p* = 0.83) was found between urban and rural preschoolers. However, significant differences were observed in sedentary time (t = −3.49, *p* < 0.01, *d* = −0.67), light-intensity physical activity (LPA; t = 3.07, *p* < 0.01, *d* = 0.62), moderate-to vigorous-physical activity (MVPA; t = 4.77, *p* < 0.01, *d* = 0.91), and all gross motor skills (*p* < 0.05). Urban children exhibited more sedentary time and lower gross motor skill scores.

**Conclusion:**

Rural preschoolers demonstrated higher physical activity levels and better gross motor skills than urban preschoolers. There is a pressing need for intervention programs to enhance physical activity and gross motor skills and reduce sedentary time among urban preschoolers.

## 1 Introduction

The prevalence of chronic diseases globally can be traced back to the early stages of human growth and development (1). Recognizing the critical importance of childhood in this context is essential, as it can provide insights into behaviors that may lead to chronic diseases later in life (2). Public health concerns regarding trends in children’s sleep, sedentary behavior, and physical activity have brought these issues to the forefront, making their resolution and reversal a strategic priority (3, 4). Physical activity (PA) is defined as any bodily movement produced by skeletal muscles that requires energy expenditure (5, 6). In contrast, sedentary behavior (SB) refers to any waking behavior characterized by an energy expenditure ≤1.5 metabolic equivalents, such as sitting, reclining, or lying down (7). Numerous correlates and determinants influence these behaviors (i.e., sedentary behavior and physical activity). Physical inactivity is often described as “the biggest public health problem of the 21st century” (8, 9), and reducing sedentary behavior is a key step toward promoting physical activity among children (3). It is well-established that engaging in PA in the early years of childhood is positively associated with various aspects of physical, psychological, social, and cognitive health (10, 11).

Increase in PA reduce the prevalence of obesity and overweight, while high levels of sedentary behavior (SB) and low levels of PA increase the risk of morbidity and mortality from cardiovascular and metabolic disorders in both childhood and adolescence (12–14). Children who engage in regular PA, experience enhanced heart and brain health, stronger muscles and bones, and a greater likelihood of maintaining a healthy weight (15, 16). These children often demonstrate better academic performance (17, 18). Research also shows that engaging in substantial PA during childhood is associated with a lower long-term risk of developing diabetes, heart disease, certain types of cancer, and anxiety (19, 20). Therefore, maintaining sufficient physical activity levels and minimizing sedentary time are crucial for preventing chronic disease morbidity and mortality (21). However, global physical activity transitions pose a potential threat to the well-being of children in Sub-Saharan Africa (SSA), where rapid sociocultural change and urbanization have shifted the economy from one based on manual labor to one dominated by industry and mechanized production (22). This shift has led to changes in habitual and occupational physical activity, moving from high-energy expenditure activities (e.g., active transport, manual labor) to low-energy expenditure activities or sedentary behaviors (e.g., motorized transport, desk work) (23, 24). The long-term health implications for children and youth in this region are particularly concerning, as they too are affected by these physical activity transitions (24).

Despite global efforts to promote school physical activity, research on preschoolers in SSA remains limited. For instance, Kidokoro et al. (25) compared physical activity and sedentary behavior among school children in Kenya and Japan, finding that Kenyan children engaged in more moderate-to-vigorous physical activity (MVPA) than their Japanese counterparts, with the largest differences observed on weekday evenings for both boys and girls and on weekend afternoons for girls. Similarly, a study found that children in Cape Town, South Africa, were sedentary 73% of the time, with low-income children spending 93% of their time indoors compared to 79% for middle-and high-income families (26). In 2020 there was an obvious changes in physical activity patterns as 96.9% of children in South Africa met WHO guidelines, with boys being more active than girls. However, children from urban high-income families were less physically active averaging 409 min of total PA, compared to their peers from urban low-income and rural low-income families, who averaged 471 and 461 min, respectively (27).

Despite being a member of the World Health Organization (WHO), Nigeria currently lacks a National Physical Activity policy for all age groups, which contrasts with WHO’s Recommendations (28, 29). Although Physical and Health Education (PHE) programs have been integrated into the primary school curriculum, it remains uncertain how effectively these programs enhance children’s PA levels. Additionally, Aoko et al. (30) found in a pilot study that 77.5% of preschoolers in Lagos Nigeria met the WHO guidelines for physical activity, with 93.5% meeting the recommended duration of moderate-to vigorous-intensity physical activity (MVPA). However, it is still unclear how geographical location influences these preschoolers’ PA patterns and interaction with health and development indices.

Therefore, this study investigated the geographic disparities in physical activity, sedentary behavior, sleep, and gross motor skills among preschoolers. We hypothesize that there will be significant differences in physical activity, sedentary behavior, sleep, and gross motor skills between preschoolers in urban and rural areas of Lagos State.

## 2 Materials and methods

### 2.1 Research design

The SUNRISE pilot cross-sectional study of preschoolers was conducted in selected urban and rural schools in Lagos state, Nigeria. A small convenience sample of 131 (3.4[SD = 0.80] years) preschoolers were drawn from the pilot phase of the SUNRISE Study. In summary, 131 healthy children were included in the gross motor skills analysis. The purpose of the study was explained to the participants and their parents before obtaining signed consent forms. The questionnaire and information sheets were completed by the parents or caregivers and school staff through interviews facilitated by a research assistant. Ethical approval for the study was obtained from the Human Research Ethics Committee at the University of Wollongong (2018/044) and the National Health Research Ethics Committee of Nigeria, with Approval Number NHREC/01/01/2007-17/01/2021.

### 2.2 Participants

The convenience stratified cluster sampling technique was used to recruit participants. Participants comprised 3.0–4.9-year-old children eligible to attend private and public preschools in two socio-economic zones (rural and urban) in Lagos State. The sample size for this study was calculated as 135 following the Sunrise study protocol published elsewhere (31) and increased to 150, to accommodate possible attritions.

### 2.3 Measures

#### 2.3.1 Physical activity, sedentary time, and sleep measure

The ActiGraph (GT3X, GT3X+) accelerometer, a waist-worn device, was used for this study. It is the most widely utilized and thoroughly validated accelerometer for assessing physical activity and sedentary behavior (31–33). Children were instructed to wear the device continuously, including during sleep but not during water-based activities such as bathing and swimming, for at least 5 days to obtain three full days (3 × 24-h periods) of data. This allowed for the collection of data on light physical activity (LPA), moderate-vigorous physical activity (MVPA), and sedentary time. Raw data were captured at 30 Hz sampling. Data was downloaded and analyzed in 15-s epochs using a low-frequency filter via ActiLife software (version 6.12.1). Participants were required to have at least 24 h of valid data (determined through a visual inspection of the acceleration data) with a minimum of 6 h of valid wear time during the waking period per day to be included in the analysis (34, 35). A predetermined time filter (based on parent-reported wake-up and bedtime) was applied to all valid 24-h day(s) to exclude sleeping periods from the analysis of physical activity and sedentary time. Waking period(s) of 20 min or more consecutive zero counts were defined as non-wear and excluded from the analysis (34, 36). Validated intensity cut points were applied to classify valid waking wear time as sedentary time (<800 counts per minute [CPM]), light-intensity physical activity (800–1,679 CPM), moderate-to vigorous-intensity physical activity (MVPA; ≥1,680 CPM), or vigorous-intensity physical activity (VPA; = > 3,368 CPM) (34, 37, 38).

Sleep was evaluated using a self-administered questionnaire (31) completed by the parents or primary caregivers. Print copies of the questionnaire were distributed, and caregivers were asked to fill them out at their convenience. Once completed, preschool teachers collected the forms and passed them on to the researchers. The following question was used to report the child’s sleep: “How many hours of sleep does this child typically get in a 24-h period, including naps? Please provide the answer as accurately as possible, rounded to the nearest minute.”

#### 2.3.2 Gross motor skills

##### 2.3.2.1 Functional mobility

The supine-timed up and go (STuG) test was used to evaluate a child’s mobility and posture. To set up the test, a line was marked 3 meters from a wall using tape, and a large target (such as a circle) was placed on the wall at the child’s eye level. The child started by lying on their back (supine) with their heels positioned on the line. Upon hearing “Go,” the child quickly got up, ran to touch the target on the wall, and then returned to cross the 3-meter line. The test included one practice trial followed by two timed trials. The timing began when the assessor said “Go” and stopped when the child’s torso crossed the starting line. The average time of the two trials was used to assess performance (31).

##### 2.3.2.2 Postural steadiness

The one-legged standing balance test was designed to assess posture and balance. The child stood on one leg with their arms relaxed at their sides for up to 30 s. The free leg was positioned off the floor, without hooking around the standing leg. Swaying was allowed, and the arms could move but could not touch the free leg. Timing started when the free leg lifted off the ground and stopped if the child moved the standing leg, hooked the free leg around the standing leg, or touched the free leg with their hands. If the child maintained balance for 30 s, the test ended. The test was then repeated on the other leg. The duration of time the child maintained balance on each leg was recorded, and the average time of both legs was used for assessment (31).

##### 2.3.2.3 Lower body strength and mobility

Children performed a standing long jump to assess their lower body explosive strength and mobility. A line was marked on the floor where the child would stand with their toes just behind it. The child was instructed to jump forward with both feet together, aiming to land as far forward as possible, also on two feet. Each child was given one practice jump followed by two test jumps. The measurement was taken from the starting line to the heel of the nearest foot upon landing, and the distance was recorded to the nearest centimeter. The average of the two test distances was used as the final score (31).

##### 2.3.2.4 Upper body strength

A handgrip dynamometer (TKK5825, Grip-A, Takei, Tokyo) was used to assess upper extremity strength. This test measures the capacity of the hand and arm muscles to produce the tension and power necessary for maintaining posture, initiating movement, or controlling movement under load. The child was required to squeeze the grip dynamometer continuously with full force using their right hand for at least 3 s without letting their arm touch their body. The test was then repeated with the left hand. The maximum measurement attained was recorded (31).

#### 2.3.3 Parent questionnaire

A parent questionnaire was used to elicit information on the child’s sociodemographic at the family level based on a modified, standardized, and validated version of the WHO STEPS survey. The child and caregiver background module comprised of 9-item questions which included the age and sex of the child. The Early Childhood Education and Care (ECEC) service questionnaire was used to determine the geographical location of the participants (31).

The researchers visited the homes and schools of the participants during the period when the accelerometers were being worn by the children to ensure their proper use. The importance of continuous wear was emphasized to the parents, and the children were encouraged to keep the devices on throughout the study period. The researchers were supported by trained field research assistants experienced in working with children. The tasks were carefully sequenced to minimize participant burden and to account for the children’s ages. Concurrently with data collection, a focus group was conducted to gather parents’ opinions on the survey questions and various aspects of the methodology.

### 2.4 Statistical analysis

Data analysis was performed using R v. 4.3.1 (R Core Team, Vienna, Austria). Normally distributed data were presented as mean ± SD, while non-normally distributed data were reported as median (interquartile range). Additionally, checks for normality and equal variance was conducted to ensure the validity of the analyses. Median imputation was applied to address missing values in gross motor skills for two participants. Independent sample t-tests were used to examine differences in physical activity (Light PA, MVPA), sedentary time, sleep, and gross motor skills (lower body strength, mobility and posture, balance, and upper body strength) between rural and urban preschoolers. Cohen’s d was used to examine the effect size. Effect size benchmarks as suggested by Cohen (39) are small (*d* = 0.2), medium (*d* = 0.5), and large (*d* = 0.8), Statistical significance was set at 0.05.

## 3 Results

**Figure 1:**
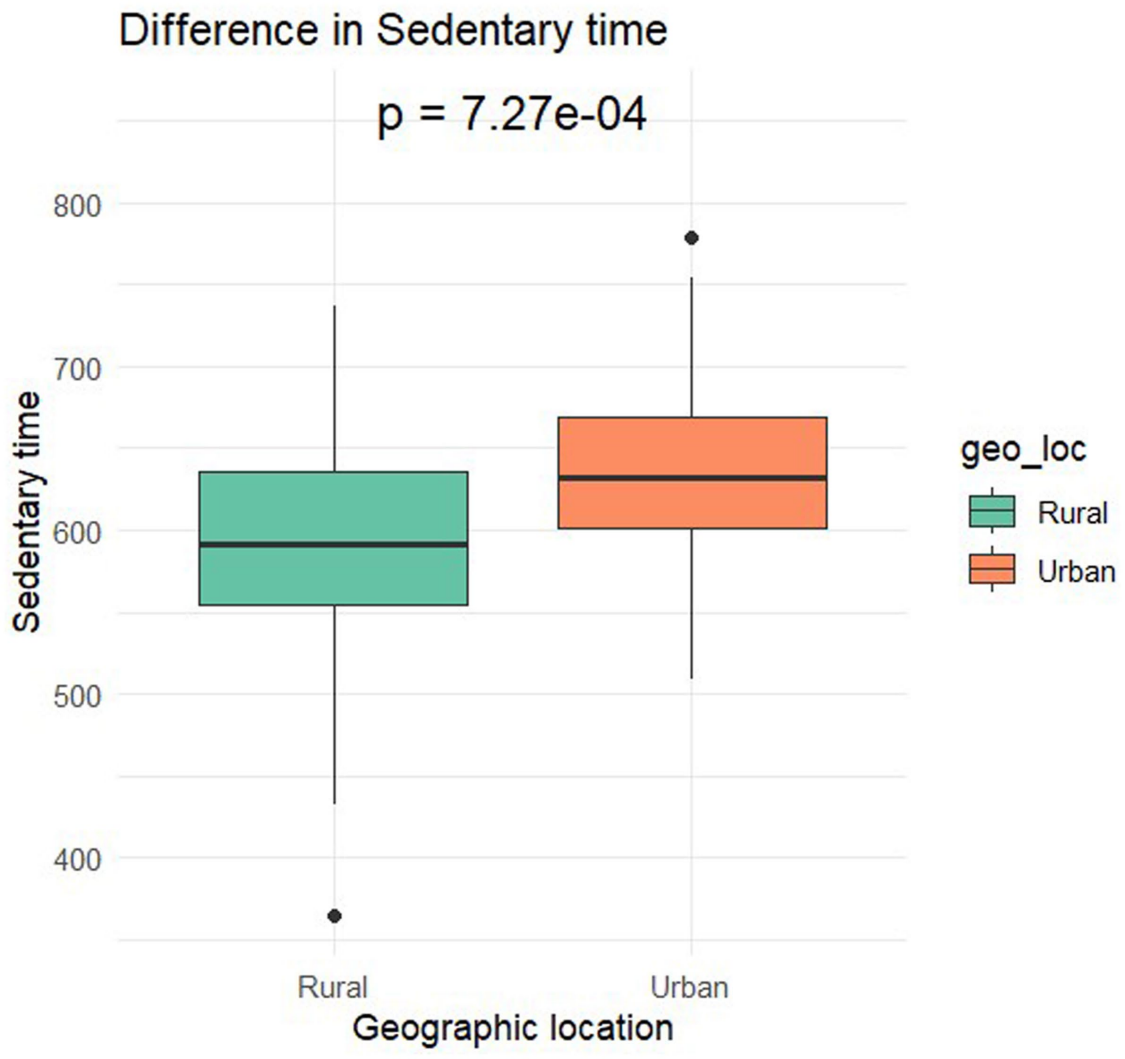
Differences in sedentary time by geographical location.

**Figure 2:**
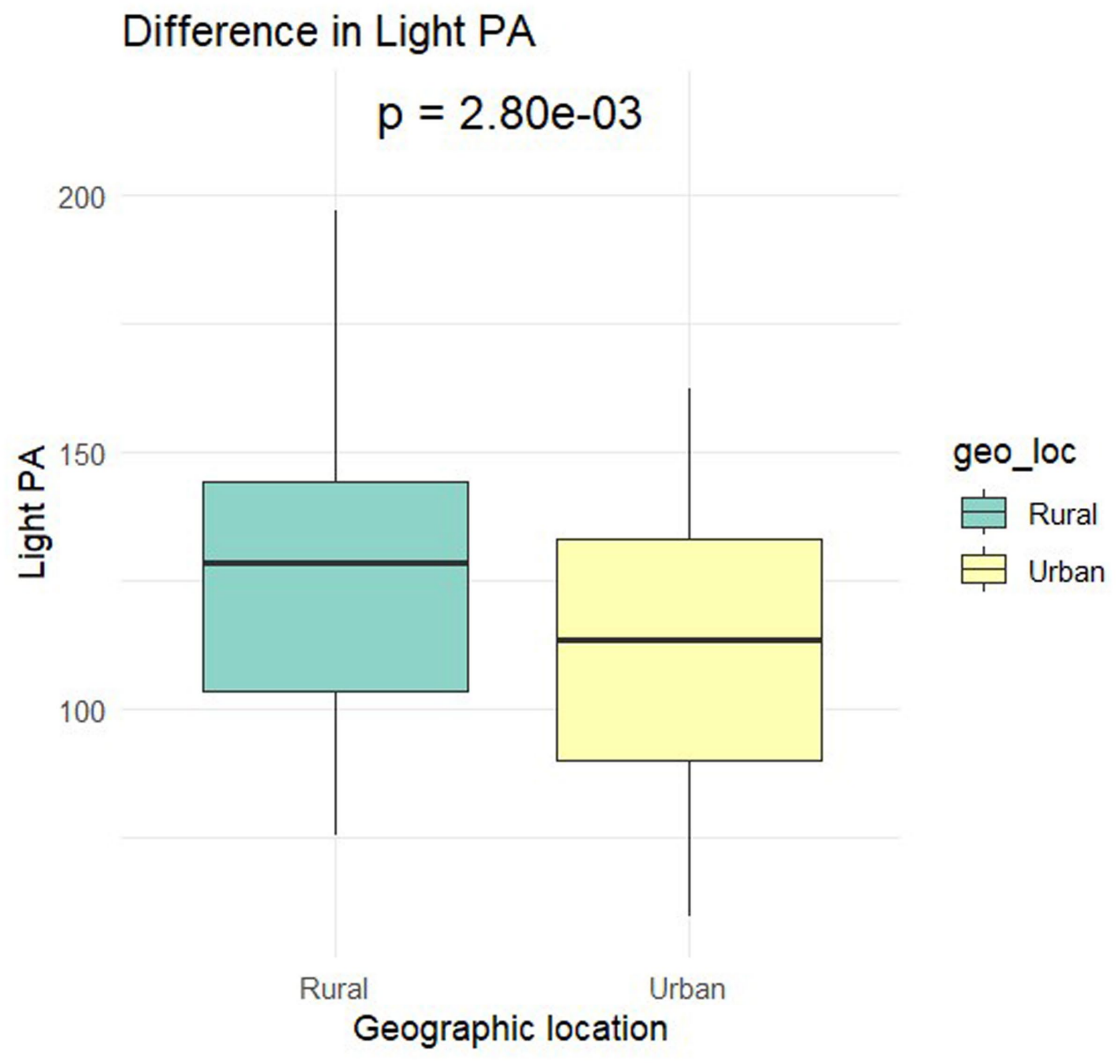
Differences in light physical activity by geographical location.

**Figure 3:**
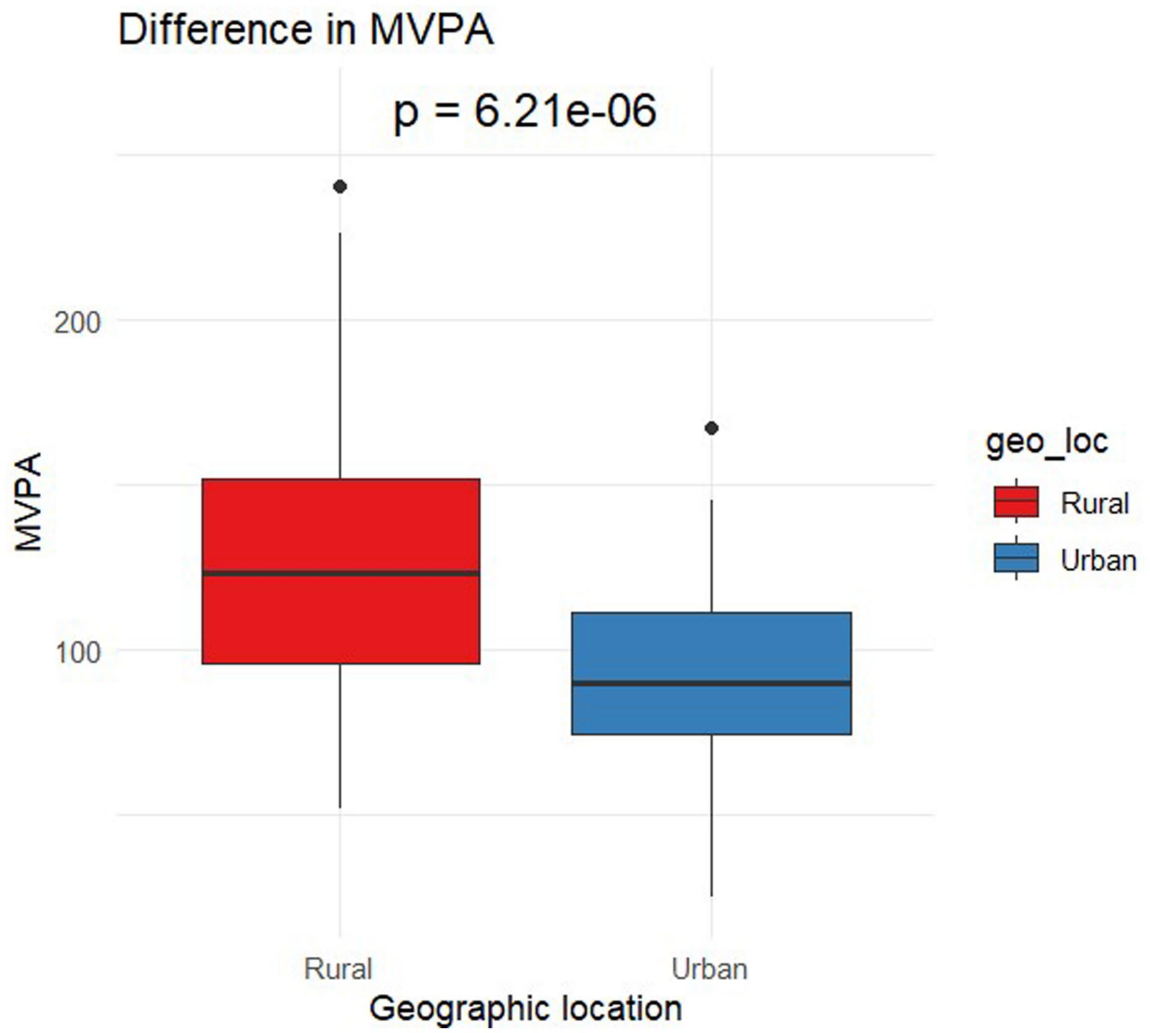
Differences in moderate-vigorous physical activity by geographical location.

Table 1 presents the demographic characteristics of the participants. The final sample comprised 131 preschoolers (M_age_ = 3.4; SD = 0.8). Overall, 56.49% were female with 43.51% male. Based on complete accelerometer data (n = 102) 58.82% were female with 41.18% male. Two-sample t-tests yielded no notable differences in parent-reported sleep duration [t(122) = 0.65, *p* = 0.52], between urban and rural preschoolers. However, significant differences were identified in Sedentary Time [t(99.6) = −3.49, *p* < 0.01] with urban preschoolers having higher sedentary time (633.87 ± 58.27 min) compared to rural counterparts (587.83 ± 75 min; Figure 1). The effect size, calculated using Cohen’s d, was-0.67, indicating a moderate effect size. In LPA [t(89.8) = 3.07, *p* < 0.01], rural preschoolers had higher LPA (127.1 ± 26.7 min) compared to urban (110.49 ± 27.1 min; Figure 2). The Cohen’s d was 0.62, indicating a moderate effect size. In MVPA [t(99.9) = 4.77, *p* < 0.01] rural preschoolers had higher MVPA (125.95 ± 41.8 min) compared to urban (92.32 ± 29.4; Figure 3). The Cohen’s d was 0.91, indicating a large effect size. No significant difference was found between boys and girls for all physical activity outcomes based on geographical location.

**Table 1:** Descriptive information.

Overall (*n* = 131)			Accelerometry sample (*n* = 102)		
Age (years)	3.4 (0.78)		Age (years)	3.4 (0.79)	
Sex	Female 56.49%	Male 43.51%	Sex	Female 58.82%	Male 41.18%
Geographical location	Rural 57.25%	Urban 42.74%	Geographical location	Rural 57.84%	Urban 42.16%
Functional mobility (5.67 [2.71])	4.64 (0.93)	7.01 (3.6)	Sedentary time (607.2 [711.9])	587.8 (75)	633.9 (58.3)
Postural steadiness (15.65 [9.4])	17.25 (9.12)	13.51 (9.44)	Light PA (120.1 [28])	127.1 (26.7)	110.5 (27.1)
Lower-body strength (68.8 [24])	74.07 (21.13)	61.85 (26.9)	MVPA (111.8 [40.5])	126 (41.8)	92.3 (29.4)
Upper-body strength^l^ (5.8 [2.7])	6.24 (2.53)	5.27 (2.87)			
Upper-body strength^r^ (6.1 [2.8])	6.51 (2.5)	5.5 (2.97)			
Sleep time (596.21 [67.82])	592.9 (69.8)	600.6 (65.5)			

MVPA = moderate-vigorous physical activity Light PA = light physical activity. l = left hand. r = right hand.

There was a significant difference in functional mobility [t(71.1) = 5.72, *p* < 0.01] with urban preschoolers having higher scores (7.01 ± 3.6) compared to rural (4.64 ± 0.93). For postural steadiness [t(129) = −2.29, *p* = 0.02], rural preschoolers had higher scores (17.25 ± 9.12) compared to urban (13.51 ± 9.12). For the lower body strength test [t(101.4) = −2.8, *p* = 0.01], rural preschoolers had higher scores (74.1 ± 21.13) compared to urban preschoolers (61.9 ± 26.9). Upper body strength assessed in the right hand [t(129) = −2.16, *p* = 0.03] revealed rural preschoolers had higher scores (6.51 ± 2.54) compared to urban (5.47 ± 2.97). While the left hand [t(129) = −2.05, *p* = 0.04] also revealed rural preschoolers had higher scores (6.24 ± 2.53) compared to urban (5.27 ± 2.87). A significant difference in functional mobility [t(64.9) = 2.15, *p* = 0.03 was found between boys and girls in rural preschoolers, with girls having higher scores (4.8 ± 1.0) compared to boys (4.38 ± 0.75). A significant difference in upper body strength assessed in the left hand [t(57.3) = −2.73, *p* = 0.01] was found between boys and girls in rural preschoolers, with boys having higher scores (7.2 ± 2.5) compared to girls (5.62 ± 2.37). While the upper body strength assessed in the right hand [t(64) = −3.11, *p* < 0.01] also revealed boys had higher scores (7.58 ± 2.26) compared to girls (5.84 ± 2.5).

## 4 Discussion

This study found that urban preschoolers in Nigeria had higher sedentary time compared to their rural counterparts. Conversely, rural preschoolers exhibited higher levels of LPA and MVPA, indicating greater overall physical activity than urban preschoolers. No significant differences in sleep duration were observed between the two groups. In terms of gross motor skills, rural preschoolers scored higher on postural steadiness, lower body strength, and upper body strength (both right and left hand). However, urban preschoolers outperformed rural preschoolers in functional mobility (STuG test). When examining sex differences, rural preschool girls scored higher in functional mobility compared to boys, while boys showed greater upper body strength in both hands than girls in rural preschools. The higher sedentary time observed in urban preschoolers is concerning, given the severe health implications of prolonged sedentary behavior, including obesity, cardiovascular disease, and metabolic disorders. Considering the negative health outcomes associated with reduced physical activity levels, targeted interventions are essential to promote active lifestyles among urban preschoolers.

The findings of this study contrast with those of McCrorie et al. (40), who reported no evidence of urban–rural differences in children’s daily MVPA or overall activity in Scotland, UK. This might be due to Scotland being a high-income country in Europe. Therefore, it is likely the differences in findings could be explained by country income levels and resources available. Although the walkability score (WS) varied between more and less deprived neighborhoods and between urban and rural areas, the study found no association between WS and children’s daily physical activity. However, a Sunrise pilot study from Tunisia Africa (41) revealed that preschoolers in rural areas have a higher percentage of compliance with the WHO integrated movement guidelines compared to preschoolers in urban areas. In line with Ltifi et al. (41) socioeconomic factors might influence this disparity which include availability and access to recreational facilities, availability of safe outdoor spaces, built environments, and the socioeconomic status of families. A study in Croatia Vukelja et al. (42), also found that children in urban settings are less active compared to those in rural areas. Additionally, Vukelja et al. highlighted that sedentary time is more prominent in urban preschoolers compared to their rural counterparts.

Our study also revealed that rural preschoolers outperformed urban preschoolers in several gross motor skills. Similar to a study from South Africa (43) that found that children from rural low-income settings performed better in gross motor skills compared to urban children. However, in terms of functional mobility, urban preschoolers surpassed rural preschoolers. Regarding sex differences, our study showed that girls in rural preschools had higher functional mobility scores compared to boys. Previous research has recognized sex differences in motor performance as an individual constraint influenced by biological factors (44, 45). However prepubertal boys and girls share similar physical attributes, such as body type, body composition, strength, and limb length (45, 46). Furthermore, girls may have demonstrated greater attentiveness to test instructions and carried out the assigned tasks with higher precision (47). However, this aligns with findings from Kwon and O’Neill (47), which indicated that girls tend to have better locomotor skills than boys. While this study was conducted in a high-income country, it potentially might rule out the influence of the overall country income status on functional mobility between boys and girls. Conversely, our study supports other research (48, 49) that concluded boys had higher upper-body strength scores compared to girls.

The findings of this study have important implications for public health policies and interventions aimed at reducing sedentary behavior, increasing physical activity levels, and enhancing gross motor skills in preschool-aged children. As mentioned in the introduction, the absence of a national physical activity policy in Nigeria complicates efforts to address these disparities. Implementing such policies could help ensure that all children, regardless of their location, have access to opportunities for physical activity. It is crucial to continue promoting gross motor skills in preschools during physical education breaks and recess. For urban preschoolers, educating parents and caregivers about the importance of encouraging gross motor activities is vital. Promoting these skills after school hours and on weekends could significantly enhance children’s gross motor skills development.

This SUNRISE Nigerian pilot study has several strengths, which comprise objective measures of physical activity, and gross motor skills, which is a novel measure of physical activity among preschoolers in Nigeria. With objective measures, the disparity uncovered necessary interventions to promote PA and gross motor skills, especially among urban preschoolers. However, this study does have some limitations. As a cross-sectional study, it reflects variations in physical activity, sedentary time, sleep, and gross motor skills within the sample rather than actual differences between individuals, which calls for a cautious interpretation of the findings. This study is a small convenience sample drawn from the pilot phase of the SUNRISE Study in Nigeria. As such, it’s not possible to generalize the data beyond this. The findings of this study might also differ considerably from a rural area located in several kilometers away from Lagos City as we differentiate the geographical location based on income status and infrastructures. Therefore, the generalizability of the results is limited. To address the issue of potential causality, longitudinal studies are needed to assess the long-term effects of early physical activity patterns on children’s health outcomes in Nigeria. In addition, a larger sample size is needed for adequate generalization to the population. The study’s findings underscore the need for locally tailored interventions in schools and homes to reduce sedentary behavior and enhance PA and gross motor skills among preschool-aged children in Nigeria. Addressing these disparities is crucial for promoting overall health and well-being from an early age and preventing the onset of chronic diseases. Future research should focus on identifying specific barriers to physical activity in urban settings and developing strategies to overcome them.

## 5 Conclusion

This study assessed and compared the physical activity, sleep behaviors, and gross motor skills of preschoolers in Nigeria, considering both geographical location and sex. It also aimed to measure the physical activity behaviors of preschoolers over a 24-h period. Significant differences were observed in the physical activities and behaviors of the children based on their location and sex. There is a need to implement strategies to encourage physical activity among children in Nigeria, with a particular emphasis on reducing sedentary behavior, especially in urban areas. Regardless of a child’s sex or residence, initiatives such as school programs, community engagement, and family involvement can play a vital role in promoting increased physical activity among young children.

## Data Availability

The raw data supporting the conclusions of this article will be made available by the authors, without undue reservation.
